# Mountain hare transcriptome and diagnostic markers as resources to monitor hybridization with European hares

**DOI:** 10.1038/sdata.2017.178

**Published:** 2017-12-05

**Authors:** João P. Marques, Mafalda S. Ferreira, Liliana Farelo, Colin M. Callahan, Klaus Hackländer, Hannes Jenny, W. Ian Montgomery, Neil Reid, Jeffrey M. Good, Paulo C. Alves, José Melo-Ferreira

**Affiliations:** 1 CIBIO, Centro de Investigação em Biodiversidade e Recursos Genéticos, InBIO Laboratório Associado, Universidade do Porto, Vairão 4485-661, Portugal; 2 Departamento de Biologia, Faculdade de Ciências do Porto, Porto 4169-007, Portugal; 3 Division of Biological Sciences, University of Montana, 32 Campus Drive, Missoula, MT 59812, USA; 4 Institute of Wildlife Biology and Game Management, BOKU-University of Natural Resources and Life Sciences, Vienna 1180, Austria; 5 Amt für Jagd und Fischerei Graubünden, Chur 7001, Switzerland; 6 Institute of Global Food Security, School of Biological Sciences, Queen’s University Belfast, Belfast BT9 5BN, UK; 7 School of Biological Sciences, Queen’s University Belfast, 97 Lisburn Road, Belfast BT9 7BL, UK

**Keywords:** Genetic markers, Transcriptomics, Conservation biology, Evolutionary genetics

## Abstract

We report the first mountain hare (*Lepus timidus*) transcriptome, produced by *de novo* assembly of RNA-sequencing reads. Data were obtained from eight specimens sampled in two localities, Alps and Ireland. The mountain hare tends to be replaced by the invading European hare (*Lepus europaeus*) in their numerous contact zones where the species hybridize, which affects their gene pool to a yet unquantified degree. We characterize and annotate the mountain hare transcriptome, detect polymorphism in the two analysed populations and use previously published data on the European hare (three specimens, representing the European lineage of the species) to identify 4 672 putative diagnostic sites between the species. A subset of 85 random independent SNPs was successfully validated using PCR and Sanger sequencing. These valuable genomic resources can be used to design tools to assess population status and monitor hybridization between species.

## Background & Summary

**Figure 1:**
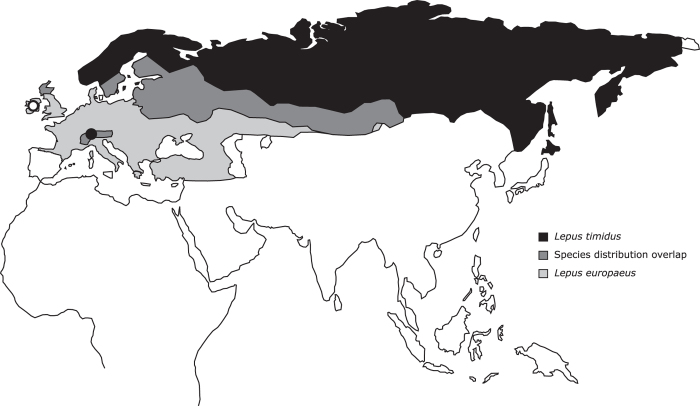
Approximate mountain and European hare distribution. Approximate distributions of the mountain hare, *Lepus timidus*, and the European hare, *L. europaeus*, in Eurasia with indication of the areas of contact and of broad geographic overlap between the species (distribution ranges were adapted from IUCN Spatial Data Resources; IUCN 2016^51^). Circles indicate the mountain hare sampling locations for this work (open circle—Ireland; closed circle—Alps).

The mountain hare (*Lepus timidus*) is an Arcto-alpine species that was the most common and widely distributed hare species across Europe during the last glacial periods^1^. Nowadays, the mountain hare is distributed from Fennoscandia to Eastern Siberia, but also occurs in isolated/refuge populations (e.g., Ireland, Scotland, the Alps, Poland, the Baltics and Japan), and in places where it has been introduced (Iceland, England, Faroe Islands and New Zealand) (see Fig. 1). Even though they are a popular game species and abundant within its range, mountain hares have sharply declined in some regions, particularly in areas of contact with the European hare (*Lepus europaeus*), where the latter tends to invade and replace the range of the former^1–4^. Mountain and European hares share extensive natural and human-induced contact zones in Western Europe, from the British Isles to Scandinavia and Central Europe (Fig. 1). Climate change is predicted to affect lagomorphs extensively^5,6^ and, in particular, to accelerate the replacement of mountain hares by European hares in the contact zones, such as the Alps, Sweden or Ireland^7,8^. The two species may hybridize when in contact, resulting in some genetic introgression^9–13^, with potential effects on local adaptation^14^.

Even though the mountain hare and other hare species have been the subject of several population genetics studies, these have been mostly based on a few markers^10,15–17^. Therefore, permanent genomic resources provide fundamental information to develop monitoring tools to evaluate population status and implement protective policies. In this work, we use high-throughput RNA sequencing to: i) generate genomic resources for the mountain hare; and, ii) use published data on the European hare^18^ to pinpoint candidate fixed differences between the species that can be used to build genotyping tools to monitor gene exchange in the contact zones. We here present the first mountain hare transcriptome, and the most complete among the currently available European *Lepus* transcriptomes.

## Methods

**Figure 2:**
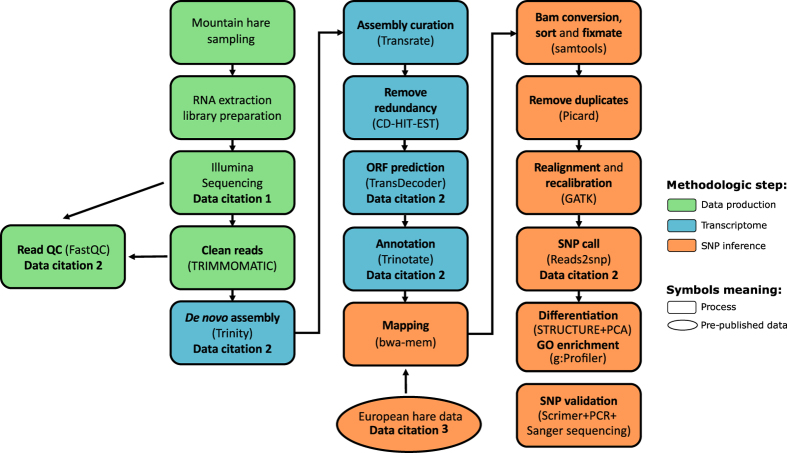
Methodological workflow. Flowchart of the RNA-sequencing setup and data analysis steps. Commands used in the analytical steps shown in bold are detailed in Table 1 (available online only).

A summary of the methodological workflow is shown in the flowchart of Fig. 2.

### Sampling procedure and locations

Specimens from the Alps (see Fig. 1) were sampled during regular permit hunting in Grisons, Switzerland. Specimens from Ireland (see Fig. 1) were captured from the wild in Borris-in-Ossory, by the Irish Coursing Club (ICC) for scientific research purposes under National Parks & Wildlife (NPWS) licence No. C 337/2012 issued by the Department of Arts, Heritage and the Gaeltacht (dated 31/10/2012). Irish hares were dispatched humanely and in accordance with the licence conditions by means of lethal injection administered by Mr William Fitzgerald, Veterinary Laboratory Service Follow (MVB MVM CertCSM), from the Department of Agriculture, Food and the Marine, Regional Veterinary Laboratory, Hebron Road, Kilkenny, R95 TX39. Total RNA was isolated from 8 individuals.

### RNA extraction

Liver tissue was freshly collected, immediately preserved in RNAlater and then stored at −80 °C until RNA extraction. Prior to extraction, frozen samples were ground in liquid nitrogen with a ceramic mortar and pestle. Mortar and pestle were washed prior to extraction using a 6-step wash that includes the following washing reagents in order: 70% ethanol, tap water, 10% bleach, milli-Q water, RNase away (Thermo Fisher Scientific) and finishing with molecular grade H_2_O. RNA extraction was performed using RNeasy Mini Kit according to manufacturer instructions.

### RNA sequencing library preparation

The SureSelect Strand-Specific RNA Library Prep for Illumina Multiplexed Sequencing (Agilent Technologies) kit was used to prepare cDNA libraries for all samples. Library sizes were estimated using a Bioanalyzer 2,100 and quantified using KAPA Library quantification kit (KAPA BIOSYSTEMS). Equal molar concentrations of each library were pooled together for sequencing.

### Sequence data processing and *de novo* transcriptome assembly

A detailed description of tools and commands used in the data analysis is shown in Table 1 (available online only). A first quality evaluation of obtained sequence reads (Data Citation 1) was performed with FastQC v0.11.5^19^. After read quality inspection, adapters were removed and quality trimming performed using TRIMMOMATIC v0.36^20^, with instructions to remove the first ten bases, Illumina adapters, reads below 25 bp long and bases in the ends of reads with quality below 10, and to perform a 4-base sliding window trimming and cutting fragments with an average quality below 10. Trimmed-read quality was rechecked with FastQC (Data Citation 2). A *de novo* transcriptome assembly was then performed using all properly paired reads from the eight individuals in the dataset using TRINITY v2.2.0^21^, establishing RF as read orientation for a strand-specific assembly. In addition, as a complementary resource, *de novo* transcriptome assemblies for each of the two sampling localities were also performed. Transrate v1.0.3^22^ was used to evaluate assembly quality and completeness and to remove possible chimeras and poorly supported contigs. Cleaned reads were mapped back to the produced assembly and only the well-supported contigs were retained (Transrate optimal cut-off >0.024). In order to remove redundancy produced by using multi-sample data to perform the assembly, all contigs were clustered using CD-HIT-EST v4.6.4^23^ with a 95% similarity threshold. Open reading frames were predicted with TransDecoder v3.0.0^24^ to remove possible contaminants such as non-coding RNA and DNA contamination. The final filtered transcriptome comprised contigs with predicted open reading frame and/or rabbit (*Oryctolagus cuniculus*) or pfam annotation. Filtered transcriptome as well as raw assemblies are available in *Figshare* (Data Citation 2).

### Transcriptome annotation

Transcriptome annotation was performed adapting the protocol of Trinotate v3.0.1^24^, using i) Conditional Reciprocal Best BLAST (crb-blast) v0.6.6^25^ against the rabbit transcriptome reference (release 86) and Swiss-Prot database^26^; ii) protein domain identification by HMMER v3.1b2^27^ onto the PFAM database^28^; iii) protein signal peptide through signal v 4.1^29^; iv) transmembrane domain prediction using tmHMM v2.0^30^; and v) eggNOG^31^, GO^32^and Kegg^33^ databases annotation. Annotation information was incorporated into an xlsx database (Data Citation 2).

### SNP inference

SNP calling was performed separately for mountain hares (Data Citation 1) and European hares (Data Citation 3, from Amoutzias *et al.*^18^). The three European hare specimens represent the European lineage of the species^18^. First, reads from all the individuals were mapped to the filtered mountain hare *de novo* transcriptome with bwa-mem v0.7.15^34^ with default parameters and read group information added to each sequencing lane-sample pair. The resulting alignments were converted to a binary file (bam format), sorted and submitted to fixmate step using SAMtools v1.3.1^35^. Duplicate reads were removed using Picard v1.140 (http://broadinstitute.github.io/picard) with the option MarkDuplicates. Realignment and recalibration was performed with Genome Analysis Toolkit v3.6-0^36^. Finally, SNP call was carried out using Reads2snp v2.0.64^37^ using a threshold of 20 for site and mapping qualities, the paralog filter, a minimum coverage of 10X and a genotype probability >0.95. The resulting VCF file was deposited in *Figshare* (Data Citation 2). Only SNPs represented in all sampled specimens were retained.

### Differentiation, admixture and Gene Ontology enrichment analysis

A set of random 5 502 SNPs, selected from independent contigs in order to reduce the linkage probability, was identified with VCFtools v0.1.14^38^. PGDSpyder v2.1.1.0^39^ was used to convert this file to the required file formats. Partitions of genetic diversity in the dataset were investigated with a Principal Components Analysis, using PLINK v1.90b3.45^40^ and ggplot2 R package^41^ to plot the results. Additionally, the data were analysed using the admixture model implemented in STRUCTURE 2.3.4^42^, with three replicate runs with 1 million steps after a burn-in period of 200 000, and K=2. Results were plotted using CLUMPACK^43^. Gene Ontology enrichment analyses were performed for the collection of contigs/genes with fixed differences between mountain and European hare samples, and between mountain hare sampling localities. The analysis was based on the rabbit proteome annotations and performed with g:Profiler^34^, applying the g:SCS multiple test correction and the ‘best per parent group’ hierarchical filter. The background set of genes was reduced to contigs with SNP information.

### Independent SNP genotyping

A random set of 110 SNPs, inferred as potentially diagnostic between *L. timidus* and *L. europaeus*, was selected for independent validation using Sanger sequencing. DNA was extracted from two of the previously analysed mountain hare samples (one Alpine, Sample_3112, and one Irish, Sample_3103) and two other European hare specimens (sampled in Clermont-Ferrand—Sample—1569—Font-Romeu, Pyrenees—Sample—1550—in France during the regular hunting season). DNA extraction was performed using JETQUICK Tissue DNA Purification kit (Genomed). PCR primers were designed to be anchored in a single exon (taking into account intron-exon boundaries from the European rabbit reference genome) and to amplify a portion of 110 independent contigs containing at least one putative diagnostic SNP. The Primer sets were designed using the Scrimer pipeline^44^, which depends on Primer3^45^ to design and set the primer conditions. A third internal sequencing primer was designed. PCRs were performed using QIAGEN Multiplex PCR Master Mix (Qiagen) and the following thermal cycling profile: initial denaturation at 95 °C for 15', 35 cycles of denaturation at 95 °C for 30'', annealing at 60–67 °C for 20'' and elongation at 72 °C for 30'', and a final extension step at 72 °C for 5'. PCR products were visually inspected under UV-light after electrophoresis in agarose gels stained with GelRed (Biotium), purified with Exonuclease I (New England Biolabs) and FastAP Thermosensitive Alkaline Phosphatase (Thermo Scientific), and sequenced using internal or, in a few cases, PCR primers in a ABI 3130xl genetic analyzer.

### Code availability

Analyses in this work were performed with freely available open access tools mainly using command line versions (Table 1 (available online only)). Parameters are described in the methods section and software versions and commands used are detailed in Table 1 (available online only).

## Data Records

Forty-eight raw FASTQ files were submitted to *NCBI Sequence Read Archive*, with accession number SRP095715 (Data Citation 1 and Tables 2 and 3). FASTQ files were divided in two sets, corresponding to the sampling localities (Ltim_Ireland and Ltim_Alps), and by biosample-specimen (SAMN06186748-3101, SAMN06186761-3102, SAMN06186762-3103 and SAMN06186763-3105; SAMN06186727-3112, SAMN06186728-3113, SAMN06186729-3114 and SAMN06186738-3116). In each biosample, six files were submitted, corresponding to three different Illumina HiSeq sequencing lanes and two read directions. Pre/post-cleaning FASTQC base quality pdf report (FASTQC.pdf) can be accessed in *Figshare* (Data Citation 2). This dataset is the core of this work and has not been released or analysed previously.

Trinity raw assemblies (Ltimidus_Trinity.fasta, LtimidusIreland_Trinity.fasta and LtimidusAlps_Trinity.fasta) were deposited on *Figshare* (Data Citation 2 and Table 4). The curated transcriptome assembly fasta files (LtimidusTranscriptome.cds.fasta and LtimidusTranscriptome.pep.fasta) and the annotated database file (LtimidusTranscriptome.xlsx) can also be found in *Figshare* (Data Citation 2).

The European hare data used here (Data Citation 3) was previously published by Amoutzias *et al.*^18^ (*NCBI Sequence Read Archive*, accession number SRP055741, samples SRR1823098, SRR1863103 and SRR1863605).

Mapping statistics (Table 5), SNP call VCF file (LtimVsLeur.vcf) and population/species diagnostic SNPs tables (Supplementary Tables 1) were deposited in *Figshare* (Data Citation 2).

## Technical Validation

### RNA integrity

The quality and quantity of each RNA sample was assessed using the 260/280 and 260/230 absorbance ratios estimated by an IMPLEN P330 NanoPhotometer and RNA Integrity Number (RIN) and concentration (μgμl^−1^) with a Bioanalyzer 2,100 (Agilent Technologies). All samples had RIN values above 8.

### RNA-Seq data quality

The Illumina HiSeq run produced a total raw output of 103 941 215 100 bp paired-end reads (207 882 430 total reads). Adapter removal and quality trimming decreased this number to 201 569 448 reads (97%) (Table 4). Final analysed reads passed the minimum quality parameters as established by FastQC.

### Transcriptome assembly curation, annotation and quality

Cleaned reads were assembled into 272 183 contigs with a mean length of 594 bp and a N50 length of 839 bp (Table 4). After assembly curation with Transrate optimal cut-off >0.024, clustering with a 95% similarity threshold and open reading frame prediction, were retained 25 868 transcripts with a mean length of 842 bp and a N50 length of 1 182 (Table 4).

**Figure 3:**
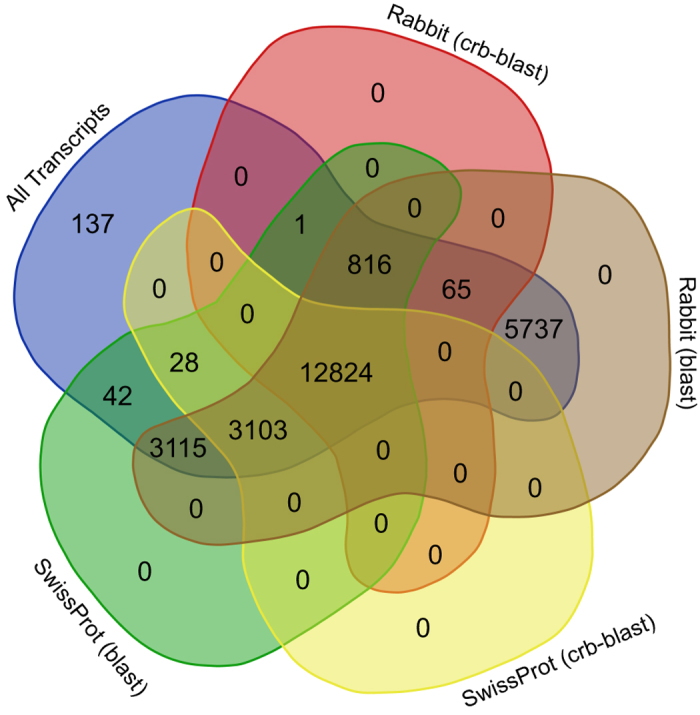
Annotation summary. Number of transcripts annotated with different combinations of methods and databases: all transcripts; transcripts annotated with crb-blast against rabbit transcriptome; transcripts annotated with a unidirectional BLASTx against rabbit transcriptome; transcripts annotated with crb-blast against the Swiss-Prot database; and transcripts annotated with a unidirectional BLASTx against the Swiss-Prot database.

Annotation using a conditional reciprocal best blast hit approach results in 16 772 (65%) annotated transcripts, of which 13 641 were annotated to the rabbit transcriptome and 15 955 to the Swiss-Prot database (Fig. 3). In order to reduce the number of non-annotated transcripts, the less stringent unidirectional blast hit was added to the database. Hits were recovered for 25 549 transcripts (99%) (Fig. 3).

The mountain hare transcriptome produced in this study represents an important improvement compared to the currently available transcriptomic resources for European *Lepus*—*L. granatensis*^46^ and *L. europaeus*^18^ transcriptomes—as it performs better on several assembly statistics, such as reference coverage (42 versus 32% in *L. granatensis* and 40% in *L. europaeus*; using the rabbit transcriptome as reference).

### Genetic variation, differentiation and gene ontology enrichment

**Figure 4:**
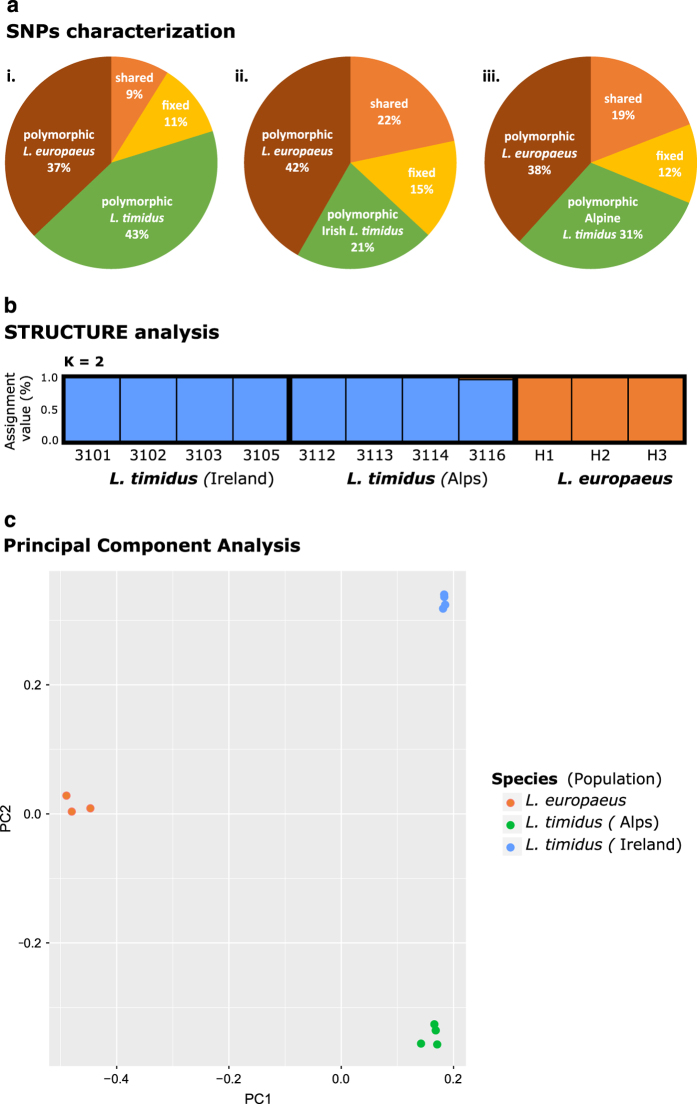
Characterization of inferred SNPs in the sampled populations and species. (**a**) Relative proportion of the 41 182 SNPs mapped to the mountain hare transcriptome, summarized as polymorphic within each species and fixed or shared between *L. timidus* (mountain hare) and *L. europaeus* (European hare). The proportion is shown considering the complete *L. timidus* dataset (i) and only the Irish (ii) and Alpine (iii) populations. (**b**) STRUCTURE analysis to evaluate cluster membership and admixture proportions. Individuals are sorted by population and species. Mountain hare populations are shown in blue and European hare individuals in orange. (**c**) Principal Component Analysis (PCA) plot using one SNP per contig. The first principal component (PC1) splits species and the second (PC2) the sampled populations.

In total, 218 057 526 reads (63%) were mapped to the filtered transcriptome—136 511 846 mountain hare reads (68%) and 81 545 680 European hare reads (57%) (see statistics in Table 5). After filtering, 159 629 high-quality SNPs were inferred, of which 41 182 (26%) were sequenced in all eleven specimens. A summary of polymorphic, shared and fixed SNPs is shown in Fig. 3. 4 672 putative species-diagnostic SNPs (considered when species presented alternative fixed alleles) were inferred (Data Citation 2,Supplementary Tables 1, also deposited in *Figshare*). The diagnostic power of our SNP set could be strongly reduced if any of the sequenced specimens was admixed (namely from the Alps, where the species overlap). We therefore conducted a Principal Component Analysis and a Bayesian Assignment analysis to assess our ability to separate the species. The results suggest that the analysed mountain and European hares are well differentiated with our SNP set, and only possible limited levels of admixture were found for Sample—3116 (Fig. 4). An extra table of putative species-diagnostic SNPs excluding that individual was therefore produced (Data Citation 2, Supplementary Table 4, also deposited in *Figshare*). 25 269 SNPs were inferred in the mountain hare, of which 12 548 and 18 591 were polymorphic in the Irish and Alpine samples respectively, and 126 were fixed between sampling localities (Data Citation 2,Supplementary Tables 5, deposited in *Figshare*). The ‘membrane part’ gene ontology term was found enriched in the collection of genes with fixed differences between the Irish and Alpine mountain hare samples, while terms ‘lipid metabolic process’, ‘small molecule catabolic process’, ‘extracellular space and acyl-CoA dehydrogenase activity’ were found enriched in genes with fixed differences between samples of the two species. Note however that even though the background gene set was controlled for, RNA-sequencing data does not provide an unbiased sample of information across different genes and these results may represent tissue-related functions.

### SNP validation

Independent SNP genotyping was performed for a random subset of 110 putative species-diagnostic SNPs from different contigs. Technical validation was considered successful for SNPs showing the expected alternative alleles, being one fixed in *L. timidus* (note that the sequenced *L. europaeus* specimens differed from the RNA-sequencing). PCR amplification was successful for 96 of the 110 target contigs (87%), 88 amplicons were successfully sequenced in both species (92%), and concordance between sequences and expected SNPs was obtained for 85 of the sequenced fragments (97%). This represents an overall validation success of 77%, which compares to studies using similar approaches^47–49^ (Data Citation 2; see Supplementary Table 8 for full genotyping results, and Supplementary Table 9 with the list of all primers, both deposited in *Figshare*). The reported accuracy of technical validation is conservative, as it is reduced by technical issues in PCR amplification and sequencing, and potential intraspecific polymorphism in the European hare (given the use of two different samples for validation), in addition to real false positives. From the validated SNPs, 73 confirmed alternate alleles in the species, but their diagnostic utility should be tested with larger population sampling.

## Usage Notes

These genomic resources (which greatly extend previously available marker sets; e.g.^50^) will be useful for a variety of studies, particularly in the characterization of genetic diversity in mountain hare populations and on the development of hybridization monitoring tools. Note that SNPs were here inferred from an uneven and small species sample, and therefore any diagnostic genotyping assay built from this data should be first tested with adequate sample sizes from pure parental populations of the species, before being applied to hybrid zones.

## Additional Information

**How to cite this article:** Marques, J. P. *et al.* Mountain hare transcriptome and diagnostic markers as resources to monitor hybridization with European hares. *Sci. Data* 4:170178 doi: 10.1038/sdata.2017.178 (2017).

**Publisher’s note:** Springer Nature remains neutral with regard to jurisdictional claims in published maps and institutional affiliations.

## Supplementary Material



## Figures and Tables

**Table 1:** Open access tools and commands used to perform data analyses (analytical steps correspond to those in Fig. 2)

Analytical Step	Description	Software/Version	Command
Read QC	Read quality control	FastQC v0.11.5	fastqc /path_to/raw.fq.gz (Data Citation 1 and Data Citation 2)
Clean Reads	Adaptor and low quality trimming	TRIMMOMATIC v0.36	java -jar /path_to/trimmomatic-0.36.jar PE -phred33 -threads 8 raw_R1.fq.gz raw_R2.fq.gz clean_FP.fq.gz clean_FU.fq.gz clean_RP.fq.gz clean_RU.fq.gz HEADCROP:10 ILLUMINACLIP:/path_to/adapters_list.fa:2:30:10 TRAILING:10 SLIDINGWINDOW:4:10 MINLEN:25
*De novo* assembly	Transcriptome assembly	Trinity v2.2.0	Trinity --seqType fq --left clean_FP.fq.gz --right clean_RP.fq.gz --CPU 20 --max_memory 150G --SS_lib_type RF --output trinity_assembly
Assembly curation	Filtering out contigs with low read support	Transrate v1.0.3	transrate --assembly Ltimidus_Trinity.fasta --left clean_FP.fq.gz --right clean_RP.fq.gz --threads 10 --reference Oryctolagus_cuniculus.OryCun2.0.81.pep.all.fa --output transrate_Ltimidus_Trinity
Remove redundancy	Clustering of highly homologous sequences	CD-HIT-EST v4.6.4	cd-hit-est -i good.Ltimidus_Trinity.fasta -c 0.95 -o AlpsIrel.fasta
ORF prediction	Filtering based on candidate coding regions and pfam annotation	TransDecoder v3.0.0	TransDecoder.LongOrfs -t AlpsIrel.fasta
		HMMER v3.1b2	hmmscan --cpu 8 --domtblout pfam.domtblout /path_to/Pfam-A.hmm transdecoder_dir/longest_orfs.pep
		TransDecoder v3.0.0	TransDecoder.Predict -t AlpsIrel.fasta --cpu 2 --retain_pfam_hits pfam.domtblout
Annotation	Annotation assessment	Trinotate v3.0.1	wget "https://data.broadinstitute.org/Trinity/Trinotate_v3_RESOURCES/Trinotate_v3.sqlite.gz" -O Trinotate.sqlite.gz
		Gunzip	gunzip Trinotate.sqlite.gz
	Conditional reciprocal best blast annotation	crb-blast v0.6.6	crb-blast --query AlpsIrel.cds --target database(SP and Ocun) --threads 4 --split 4 --output blastx.outfmt6
		crb-blast v0.6.6	crb-blast --query AlpsIrel.pep --target database(SP and Ocun) --threads 4 --split 4 --output blastp.outfmt6
	Signalp annotation	signalp v4.1	signalp -f short -n signalp.out AlpsIrel.pep
	Pfam annotation	HMMER v3.1b2	hmmscan --cpu 2 --domtblout TrinotatePFAM.out Pfam-A.hmm AlpsIrel.pep
	tmhmm annotation	tmHMM v2.0	tmhmm --short < AlpsIrel.pep > tmhmm.out
	Combine annotations	Trinity utilities v2.2.0	/path_to/trinityrnaseq-2.2.0/util/support_scripts/get_Trinity_gene_to_trans_map.pl AlpsIrel.fasta >AlpsIrel.gene_trans_map
		Trinotate v3.0.1	Trinotate Trinotate.sqlite init --gene_trans_map AlpsIrel.gene_trans_map --transcript_fasta AlpsIrel.fasta --transdecoder_pep AlpsIrel.pep
	SwissProt annotation load	Trinotate v3.0.1	Trinotate Trinotate.sqlite LOAD_swissprot_blastp SP.blastp.outfmt6 #and# Trinotate Trinotate.sqlite LOAD_swissprot_blastx SP.blastx.outfmt6
	*O.cuniculus* annotation load	Trinotate v3.0.1	1. Trinotate Trinotate.sqlite LOAD_custom_blast --outfmt6 Ocun.blastp.outfmt6 --prog blastp --dbtype Ocun; 2. Trinotate Trinotate.sqlite LOAD_custom_blast --outfmt6 Ocun.blastx.outfmt6 --prog blastx --dbtype Ocun
	Pfam annotation load	Trinotate v3.0.1	Trinotate Trinotate.sqlite LOAD_pfam TrinotatePFAM.out
	tmhmm annotation load	Trinotate v3.0.1	Trinotate Trinotate.sqlite LOAD_tmhmm tmhmm.out
	Signalp annotation load	Trinotate v3.0.1	Trinotate Trinotate.sqlite LOAD_signalp signalp.out
	Joint annotation file	Trinotate v3.0.1	Trinotate Trinotate.sqlite report > LtimidusTranscriptome.xls
Mapping	Read mapping onto the curated reference	bwa-mem v0.7.15	bwa index AlpsIrel.cds
		bwa-mem v0.7.15	bwa mem -t 10 -R '@RG\tID:pop_sample_lane\tSM:popsample\tLB:LIBsample' AlpsIrel.cds Sample_L*_FP.fq.gz Sample_L*_RP.fq.gz > Sample_lane.sam
Bam conversion,sort and fixmate	Fixmate and BAM conversion	SAMtools v1.3.1	samtools fixmate --output-fmt BAM sample_lane.sam sample_lane_fixmate.bam
	BAM sort	SAMtools v1.3.1	samtools sort -O bam -o sample_lane_sorted.bam -T /path_to/temp/ sample_lane_fixmate.bam
Remove duplicates	Mark and remove duplicates	Picard v1.140	java -jar /path_to/picard.jar MarkDuplicates REMOVE_DUPLICATES=True MAX_FILE_HANDLES_FOR_READ_ENDS_MAP=950 ASSUME_SORTED=true VALIDATION_STRINGENCY=SILENT I=sample_lane_sorted.bam I=sample_lane_sorted.bam I=sample_lane_sorted.bam O=sample_rmdup.bam M=duplic_stats_sample TMP_DIR=/path_to/temp
Realignment and recalibration	Realignment	GATK v3.6-0	java -jar /path_to/GenomeAnalysisTK.jar -T RealignerTargetCreator -R AlpsIrel.cds -I sample_rmdup.bam -o sample_int.list
	Recalibration	GATK v3.6-0	java -jar /path_to/GenomeAnalysisTK.jar -T IndelRealigner -R AlpsIrel.cds -I sample_rmdup.bam -targetIntervals sample_int.list -o sample_realign.bam
SNP call	SNP call	Reads2snp v2.0.64	reads2snp_2.0.64.bin -bamlist LtimLeur_list.txt -bamref AlpsIrel.cds -out LtimVsLeur -min 10 -nbth 12 -th1 0.95 -par 1 -th2 0.01 -opt bfgs -fis 0.0 -pre 0.001 -rqt 20
Differentiation analysis	Remove indels and missing data	VCFtools v0.1.14	vcftools --vcf LtimVsLeur.vcf --recode --recode-INFO-all --remove-indels --max-missing-count 0 --out LtimVsLeur_noindels
	Extract 1 SNP per contig	VCFtools v0.1.14	vcftools --vcf LtimVsLeur_noindels.recode.vcf --recode --recode-INFO-all --thin 10000 --min-alleles 2 --out LtimVsLeur_1SNPperContig
	VCF to STRUCTURE conversion	PGDSpyder v2.1.1.0	java -Xmx1024m -Xms512m -jar /path_to/PGDSpider2-cli.jar -inputfile LtimVsLeur_1SNPperContig.recode.vcf -inputformat VCF -outputfile LtimVsLeur_SNPs -outputformat STRUCTURE -spid VCF_to_STRUCTURE.spid
	Structure analysis	STRUCTURE v2.3.4	structure -m mainparams (standard parameters except 1 million steps after a burn-in period of 200 000, K=2 and admixture model)
		CLUMPACK v42089	The Web version was used - http://clumpak.tau.ac.il/
	PCA analysis	PLINK v1.90b3.45	plink --file LtimVsLeur_1SNPperContig --pca 3
		ggplot2 R package v2.2.1	1. R; 2. library(ggfortify); 3. pca <- read.table('plink.eigenvec', header=TRUE); 4. df <- pca[c(3, 4)]; 5. autoplot(prcomp(df), data=pca, colour='Species.Pop', size=5)
GO enrichment	Gene Ontology enrichment analysis	g:Profiler	Available at http://biit.cs.ut.ee/gprofiler/ ; Best per parent group Hierarchical filtering; Input background manually; g:SCS significance threshold.

**Table 2:** Summary of sample data information deposited in the NCBI database.

**Sample ID**	**Species (population)**	**Tissue**	**Method**	**NCBI BioSample ID**
Sample_3101	*Lepus timidus* (Ireland)	liver	RNA-seq	SAMN06186748
Sample_3102	*Lepus timidus* (Ireland)	liver	RNA-seq	SAMN06186761
Sample_3103	*Lepus timidus* (Ireland)	liver	RNA-seq	SAMN06186762
Sample_3105	*Lepus timidus* (Ireland)	liver	RNA-seq	SAMN06186763
Sample_3112	*Lepus timidus* (Alps)	liver	RNA-seq	SAMN06186727
Sample_3113	*Lepus timidus* (Alps)	liver	RNA-seq	SAMN06186728
Sample_3114	*Lepus timidus* (Alps)	liver	RNA-seq	SAMN06186729
Sample_3116	*Lepus timidus* (Alps)	liver	RNA-seq	SAMN06186738

**Table 3:** Illumina RNA-seq data deposited in the NCBI database.

**Sample ID**	**NCBI SRA runs accession**	**Raw reads**	**Mbytes**
Sample_3101	SRR5133282	26,598,712	2,525
Sample_3102	SRR5133280	26,128,525	2,532
Sample_3103	SRR5133285	24,469,456	2,414
Sample_3105	SRR5133283	26,662,182	2,582
Sample_3112	SRR5133287	22,444,667	2,263
Sample_3113	SRR5133281	20,825,930	2,100
Sample_3114	SRR5133286	32,749,011	3,294
Sample_3116	SRR5133284	21,690,965	2,189

**Table 4:** Mountain hare transcriptome assembly and curation statistics.

***Lepus timidus*** **transcriptome**	**Value**
Raw Reads	207,882,430
Clean Reads	201,569,448
Mapped Reads	136,511,846
Raw *de novo* assembly (Trinity)	
Number of contigs	272,183
Largest (bp)	14,048
Smallest (bp)	201
N50 (bp)	839
Mean (bp)	594
Post assembly curation (TransRate)	
Number of contigs	113,694
Largest (bp)	14,048
Smallest (bp)	201
N50 (bp)	801
Mean (bp)	567
Post redundancy removal (CD-HIT-EST)	
Number of contigs	109,239
Largest (bp)	14,048
Smallest (bp)	201
N50 (bp)	765
Mean (bp)	554
Post open reading frame prediction (TransDecoder)	
Number of contigs	25,868
Largest (bp)	13,728
Smallest (bp)	297
N50 (bp)	1,182
Mean (bp)	842
Reference Coverage (%)	42

**Table 5:** Mapping statistics.

**Sample ID**	**Species (population)**	**Raw reads #**	**Mapped reads #**	**Mapped reads %**
Sample_3101	*Lepus timidus* (Ireland)	26,598,712	19,648,435	74
Sample_3102	*Lepus timidus* (Ireland)	26,128,525	18,781,893	72
Sample_3103	*Lepus timidus* (Ireland)	24,469,456	16,102,091	66
Sample_3105	*Lepus timidus* (Ireland)	26,662,182	18,429,333	69
Sample_3112	*Lepus timidus* (Alps)	22,444,667	13,913,982	62
Sample_3113	*Lepus timidus* (Alps)	20,825,930	13,935,177	67
Sample_3114	*Lepus timidus* (Alps)	32,749,011	21,360,771	65
Sample_3116	*Lepus timidus* (Alps)	21,690,965	14,340,164	66
Sample_H1	*Lepus europaeus*	20,825,930	14,100,961	62
Sample_H2	*Lepus europaeus*	32,749,011	28,922,352	57
Sample_H3	*Lepus europaeus*	21,690,965	38,522,367	55
